# Fibrillary glomerulonephritis with small fibrils in a patient with the antiphospholipid antibody syndrome successfully treated with immunosuppressive therapy

**DOI:** 10.1186/1471-2369-8-7

**Published:** 2007-05-09

**Authors:** Muhammad M Javaid, Helen Denley, Senyo Tagboto

**Affiliations:** 1 Glan Clwyd Hospital, Bodelwyddan, Rhyl, LL18 5UJ, UK; 2 Department of Histopathology, 1st Floor, Clinical Sciences Building 1, Central Manchester and Manchester Childrens University Hospitals NHS Trust, Oxford Road, Manchester, M13 9WL, UK; 3 University Hospital of North Staffordshire, Royal Infirmary, Princes Road, Hartshill, Stoke-on-Trent, ST4 7LN, UK

## Abstract

**Background:**

Fibrillary glomerulonephritis is a rare cause of progressive renal dysfunction, often leading to the need for dialysis within a few years. The role of immunosuppressive treatment is still uncertain although this has been tried with variable success.

**Case presentation:**

A 56 year old woman with the antiphospholipid antibody syndrome (IgM anticardiolipin antibodies) was seen in the nephrology clinic with haematuria, proteinuria, and worsening renal function. A renal biopsy demonstrated a mesangial proliferative glomerulonephritis on light microscopy and smaller fibrils (10.6–13.8 nm in diameter) than is usual for fibrillary glomerulonephritis (typically 18–22 nm) on electron microscopy. Amyloidosis was excluded following detailed evaluation. On account of rapidly worsening renal failure she was started on cyclophosphamide and prednisolone which led to the partial recovery and stabilization of her renal function.

**Conclusion:**

This case highlights the need for routine electron microscopy in native renal biopsies, where the differential diagnosis is wide and varied and the light and immunofluorescence microscopic findings may be non specific.

## Background

Fibrillary glomerulonephritis (FibGN) is a rare cause of progressive renal dysfunction. The majority of patients who develop the disease require dialysis within a few years [1]. It was first described by Rosenmann and Eliakim in 1977 as an amyloid-like glomerulopathy but with negative congo red staining [2]. Alpers *et al *introduced the term FibGN in 1987 [3]. It is characterized by the deposition of randomly arranged fibrils in the mesangium and glomerular basement membrane. The fibrils are generally less than 30 nm in diameter, with the majority measuring approximately 20 nm. This condition is closely related to immunotactoid glomerulopathy (see table 1) [4-8]. There is some overlap between these two conditions, which has led some pathologists to propose that they should be classified together as one entity [9].

**Table 1:** Classification and clinical features of fibrillary and immunotactoid glomerulopathies

	**Fibrillary glomerulonephritis**	**Immunotactoid glomerulopathy**
Composition	Fibrils	Microtubules
Fibril or microtubule size	Average diameter 18–22 nm (usual range 12–30 nm)	Typically >30 nm (range 16–90 nm)
Arrangement of fibrils or microtubules	Randomly arranged fibrils	Parallel arrays of microtubules
Immunoglobulin type	Usually polyclonal (mostly IgG4 sometimes with IGg1) occasionally monoclonal (IgGκ)	Usually monoclonal IgGκ or IgGλ
Light microscopy	Mesangial proliferation, membranoproliferative GN crescentic GN, sclerosing GN diffuse proliferative GN with endocapilliary exudation	Atypical membranous GN, diffuse proliferative GN membranoproliferative GN
Association with lymphoproliferative disorder	Uncommon	Common (chronic lymphocytic leukaemia, nonHodgkin lymphoma)
Renal presentation	Sub nephrotic or nephrotic range proteinuria + haematuria hypertension, rapidly progressive glomerulonephritis	Nephrotic syndrome with microhaematuria and hypertension
Other manifestations (fibrillar deposits)	Pulmonary haemorrhage	Microtubular inclusions in leukaemic lymphocytes
Treatment	Various immunosuppressive drugs tried with variable response (see table 1)	Treatment of the associated lymphoproliferative disorder
Racial predilection	Predominantly Caucasian	Predominantly Caucasian
Peak occurrence	5th to 6th decades	Age 60 years
Prognosis	Established renal failure in half of patients within 2–4 years	Probably better renal prognosis than fibrillary GN
Frequency in renal biopsies	Approximately 1 % of renal biopsies	0.06% of renal biopsies

Light microscopy typically demonstrates a mesangioproliferative or a membranoproliferative glomerulonephritis. Glomerular crescents are present in about 25% of biopsies [1,10]. Immunofluorescence may demonstrate IgG and C3, IgG4 being the predominant IgG subtype [5,6]. IgA, IgM and C1q deposition are less commonly found.

We report a case of FibGN in a 56 year old woman. The size of her fibrils were rather small ranging between 10.6–13.8 nm. Further detailed evaluation did not demonstrate amyloid deposition. On account of rapidly worsening renal failure she was started on a trial of cyclophosphamide and prednisolone which led to the partial recovery and stabilization of her renal function.

## Case Presentation

A 56 year old woman was referred to the nephrology outpatient clinic, in November 2004 with haematuria, proteinuria, and worsening renal function. Her only complaints were of intermittent macroscopic haematuria and right upper quadrant colicky abdominal pain.

Her past medical history included hypertension, hyperlipidaemia and psoriasis. Additionally, she had an appendicectomy aged 16 and a cholecystectomy in 1984. She had been diagnosed with the antiphospholipid antibody syndrome (IgM anticardiolipin antibodies) following an episode of branch retinal artery thrombosis in September 2003, and a transient ischaemic attack in January 2004.

Her medications included warfarin, atorvastatin and perindopril, although the latter had just been stopped by her General Practitioner.

At the time of her initial review in the renal out-patient clinic, her blood pressure was 164/90 mmHg. Her urine dipstick contained blood (+++) and protein quantified at 0.52 g in 24 hours. Serum albumin levels were low at 31 g/l. An ultrasound scan demonstrated normal kidneys with a small benign cyst on her left kidney. An IVU and cystoscopy were reported as normal. Her serum creatinine levels measured 84 μmol/l in July 2003, 150 μmol/l in November 2004, and 300 μmol/l in January 2005. Further investigations showed a haemoglobin level of 8 g/dl. Serum Folate levels were normal; however, B12 and Ferritin levels were low at 171 pg/ml and 33.7 ng/ml respectively. Gastric parietal cell antibodies and intrinsic factor antibodies were not detected.

ANA, ENA, ANCA and dsDNA antibodies, rheumatoid factor and cryoglobylins were not demonstrated. Serum angiotensin converting enzyme (ACE) levels and complement (C3 & C4) were within normal limits.

Serology for Hepatitis B and C was negative. C reactive protein levels were measured at 4 mg/l. Her ESR and plasma viscosity were elevated at 76 mm/hr and 1.98 mPas respectively. Serum electrophoresis revealed no abnormalities, and Bence-Jones proteins were not detected in a urine specimen.

**Figure 1:**
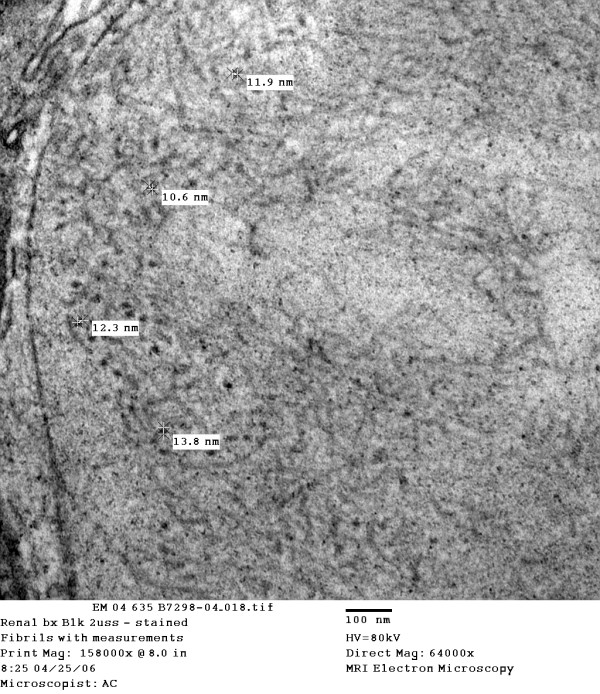
Electron micrograph or kidney biopsy demonstrating fibrils ranging in size from 10.6 to 13.8 nm (original magnification × 64000).

**Figure 2:**
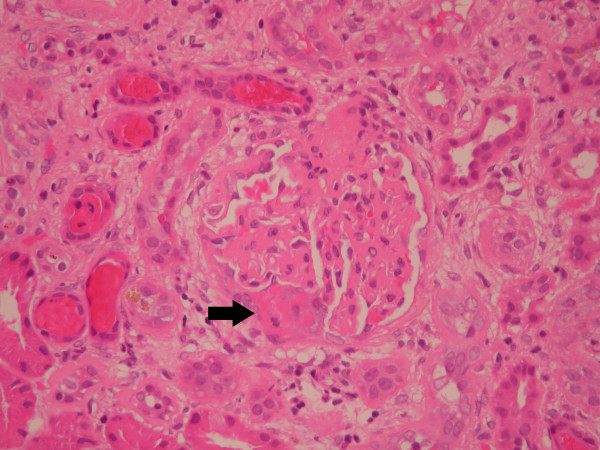
Kidney biopsy showing possible early crescent (haematoxylin and eosin stain, original magnification × 100).

**Figure 3:**
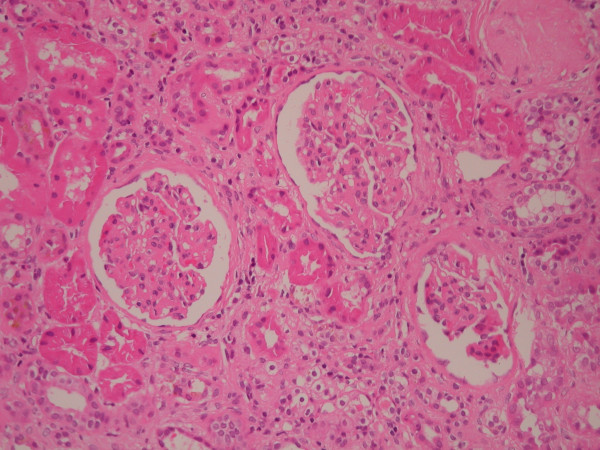
Kidney biopsy showing mesangial proliferative changes (haematoxylin and eosin stain, original magnification ×100).

**Figure 4:**
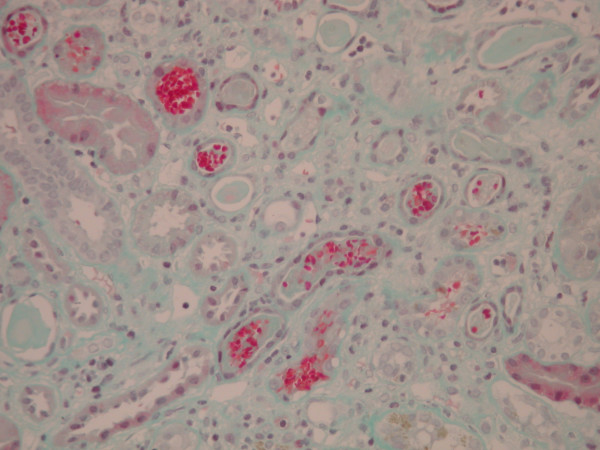
Red cells and red cell cast in tubules (Masson trichrome stain, original magnification ×100).

Light microscopy showed nine glomeruli, two of which were globally sclerosed. Of the remainder, many showed a mesangial matrix increase with hypercellularity, without the evidence of necrotizing lesions or fibrin thrombi (fig. 1). Silver stain showed basement membrane wrinkling, but no evidence of spikes or double contours. In a single glomerulus on one level, there was prominence of the epithelial cells of Bowman's capsule, possibly with some associated fibrin possibly representing an early crescent (fig. 2). Established crescents were not seen. There was a moderate and patchy interstitial inflammatory infiltrate. There was no interstitial fibrosis or evidence of tubular atrophy. Some tubules contained red cells (fig. 3). The arteries showed focal thickening of their walls. Immunofluorescence showed diffuse patchy granular mesangial and peripheral IgG (2+), C3 (+), and IgM (+) (fig 4). IgA and C1q were negative. The appearance was essentially of a mesangial proliferative glomerulonephritis. Congo red stain was negative. Immunohistochemistry for AA, AL and Aβ2M amyloid were negative.

Electron microscopy of a single glomerulus showed numerous fibrils with a diameter of 10.6–13.8 nm and within an expanded mesangial matrix. These findings suggested the diagnosis of FibGN although the small fibre size was somewhat suggestive of amyloid (FibGN typically has larger fibers, average diameter 20 nm).

She was referred to the National Amyloidosis Centre who also assessed her renal biopsy and did not find evidence of amyloid. They assessed her serum free light chains, demonstrating slightly increased kappa levels at 29.3 mg/l and lambda levels at 39.7 mg/l, probably the consequence of the decreased renal clearance of normal light chains in this patient. However, the κ/λ ratio was normal at 0.74 (range 0.26–1.65). Furthermore, serum amyloid P (SAP) scintigraphy did not reveal visceral amyloid deposition. A final diagnosis of FibGN was made.

Once her serum creatinine levels reached 300 μmol/l in January 2005, she was started on Prednisolone (40 mg daily) and cyclophosphamide (100 mg daily). In addition she received intravenous iron therapy, B12 injections and erythropoietin beta. Her blood pressure was treated with perindopril and amlodipine.

**Figure 5:**
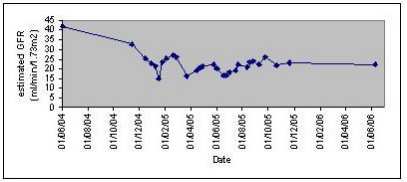
Changes in estimated GFR (abbreviated MDRD formula) with time (see text for details).

Following the initiation of immunosuppressive treatment, her serum creatinine levels improved to178 μmol/l in four weeks. Cyclophosphamide treatment was briefly withheld on two occasions in March and in May 2005 due to various minor infections, during which time her serum creatinine increased to 280 and 270 μmol/l respectively. Creatinine levels again improved on restarting immunosuppressive treatment to levels of approximately 200 μmol/l. Her renal function remained stable subsequently. Over the next year of follow, the dose of her cyclophosphamide was maintained while her prednisolone was slowly reduced to 5 mg daily. Following discontinuation of immunosuppressive treatment after a year, serum creatinine levels remained stable over the next 6 months of follow up (fig. 5).

During the course of her illness, she had an episode of haematemisis and fresh bleeding per rectum. She also complained of easy skin bruising and had an episode of macroscopic haematuria. Upper gastrointestinal endoscopy and colonoscopy were normal. Unfortunately, a request, with the patient's permission to obtain biopsies at endoscopy to look for fibrils was inadvertently not carried out. A chest X-ray carried out to investigate a cough was reported as showing a bulky left hilum but a computed tomography scan of chest and upper abdomen was normal.

FibGN occurs in approximately 1% of renal biopsies [[8,11] and [12]]. It is more common in Caucasians with a peak incidence between the fifth and the sixth decades of life [11].

Since the patient had been diagnosed with the antiphospholipid antibody syndrome, the presence of mesangioproliferative glomerulonephritis on light microscopy, initially suggested the diagnosis of lupus nephritis. However the presence of fibrils in the mesangium on electron microscopy and the negative staining for amyloid pointed to a diagnosis of FibGN, a condition with a relatively poor prognosis. It is quite possible that the diagnosis may be underreported where light microscopic findings are not routinely complemented by electron microscopy.

The fibrils in our patient were 10.6–13.8 nm in diameter. Fibrils of this size are possible in amyloidosis, which is characterized by the deposition of proteinaceous material in extracellular spaces, composed of a felt like array of 7.5–10 nm wide, linear, non branching fibrils of indefinite length [13]. In our patient, congo red staining, immunohistochemistry and SAP scintigraphy did not reveal amyloid.

Symptoms in FibGN are usually nonspecific and consist of proteinuria, haematuria, hypertension and renal insufficiency. Fibril deposition is predominantly confined to the kidneys. However, fibrillary deposits have been reported in the alveolar capillary membrane, producing a pulmonary-renal syndrome, and in the skin of a patient with leukocytoclastic skin vasculitis [7,12,14]. Whether the episodes skin bruising, haematemesis, and rectal bleeding in our patient was due to fibrillary deposition in these organs or not is not known. Our patient was understandably not keen to be subjected to further biopsies to confirm the deposition of fibrils elsewhere.

The pathogenesis of FibGN is not clearly understood at present. Immunofluorescence findings suggest an immunogenic origin. There in no definitive treatment for this condition. A few cases have been reported in the literature where immunosuppressive treatment has been used with varying success (see table 2) [15-32]. Half of all cases develop end stage renal failure within 4 years of diagnosis [31]. Although recurrence after renal transplantation has been described, the progression of renal dysfunction is often slow [31]. In our patient prednisolone and oral cyclophosphamide was started due to moderately rapidly deteriorating renal function. Renal function improved substantially within four weeks of initiating treatment and remained stable to date after more than a year of follow up. It is interesting to note that early in the course of her illness, treatment was briefly stopped on two occasions during which her serum creatinine levels increased.

**Table 2:** Key publications regarding the efficacy of immunosuppressive drugs in treating fibrillary glomerulonephritis

**Treatment**	**Response**	**Authors**
Methyprednisolone/Prednisolone	No response	*Asaba et al., 2003*
Prednisolone & Mycophenolate mofetil	Persistent haematuria and proteinuria, improvement in serum creatinine levels	*Bijol et al., 2006*
Prednisolone, Cyclophosphamide then Cyclosporin	Brief initial response with prednisolone then relapse. No response with cyclophosphamide resolution of nephrotic syndrome with cyclosporin	*Bircan et al., 2004*
Prednisolone & Cyclophosphamide	Marked improvement in renal function	*Blume et al., 2002*
Corticosteroids +/- Cyclophosphamide	No response in fibrillary glomerulonephritis. Variable response in immunotactoid glomerulopathy including melphalan, vincristine, doxorubicin, carmustine or chlorambucil in various combinations, remission or improvement in nephrotic syndrome in 10/12 patients, disappearance of deposits from the kidneys of 2 patients	*Bridoux et al., 2002*
Prednisolone & Cyclophosphamide then Azathioprine	Transient reduction in proteinuria	*Chan han, 1998*
Prednisolone	Resolution of nephrotic syndrome or reduction in proteinuria	*Dickenmann et al., 2002*
Prednisolone & Chlorambucil	Slight reduction in creatinine, reduction in proteinuria from 3 g to 1 g in 24 h.	*Dussol et al., 1998*
Prednisolone	No response	*Gielen et al., 2006*
Prednisolone & Cyclophosphamide then Azathioprine	No response	*Hsu et al., 2001*
Prednisolone & Cyclophosphamide	Recovery of renal function, discontinuation of dialysis	*Mahajan et al., 2005*
Prednisolone	Improvement in renal function, reduction in proteinuria	*Moriyama et al., 2003*
Prednisolone & Chloraminophen (Chlorambucil)	Reduction in creatinine levels, disappearance of proteinuria and haematuria	*Nabarra et al., 2003*
Prednisolone & Mycophenolate mofetil	Continued rapid deterioration in renal function	*Ovuworie et al., 2000*
Prednisolone, Cyclophosphamide, plasmapheresis & immunoglobulins	Fibrils in renal transplant patient, continued rapid deterioration in renal function	*Palanichamy et al., 1998*
Various combinations of Cyclosporin A	Biopsy proven recurrence of fibrillary glomerulopathy in 3 transplanted kidneys	*Pronovost et al., 1996*
Prednisolone, Azathioprine and ATG (in 1 patient)	Rate of decline in renal allografts slower than native kidneys suggesting either benefit of immunosuppressive therapy or spontaneous remission with time	
20 treated patients out of 56	No effect of immunosuppression on incidence of or time to end stage renal disease	*Rosenstock et al., 2003*
Steroids alone (16% of patients)	Variable response including improvement in renal function and reduction in proteinuria	
Cyclophosphamide +/- steroids (14%) Cyclosporin (5%)	Poor response of patients with immunotactoid glomerulopathy to immunosuppression except one good responder to fludarabine with improvement in renal function and reduced proteinuria	
Prednisolone & Cyclophosphamide	Recovery of renal function, discontinuation of dialysis, recovery from pulmonary haemorrhage	*Rovin et al., 1993*
Prednisolone & Chlorambucil	Reduction or stabilization in creatinine levels, reduction in proteinuria	*Schneider et al., 1996*

## Conclusion

This case highlights the need for routine electron microscopy in native renal biopsies, where the differential diagnosis is wide and varied and the light and immunofluorescence microscopic findings may be non specific.

The diagnosis of FibGN cannot be made on the basis of the size of the fibrils alone. A therapeutic trial of cyclophosphamide and prednisolone in patients with progressive renal dysfunction who are able to tolerate the treatment, and who understand the risks is worthwhile. This may prevent or slow down the progression of renal failure.

## Competing interests

The author(s) declare that they have no competing interests.

## Authors' contributions

MMJ was involved in drafting and revising the original manuscript. HD reported the renal biopsy findings and was involved in revising the manuscript. ST was the consultant responsible for the management of this patient. He was involved in critically revising the manuscript. All the authors read and approved the final manuscript.

## Pre-publication history

The pre-publication history for this paper can be accessed here:


